# The learning curve for the Shouldice Repair: a pilot analysis of post-training specialized surgeons at the Shouldice Hospital

**DOI:** 10.1007/s10029-024-03252-0

**Published:** 2025-01-23

**Authors:** Christoph Paasch, Richard Hunger, Peter Szasz, Ayse Yilbas, Fernando A. C. Spencer Netto, Rene Mantke, Marguerite Mainprize

**Affiliations:** 1 Department of Surgery, Shouldice Hospital, Markham, ON Canada; 2 Department of General Surgery, University Hospital Brandenburg an der Havel, Brandenburg Medical School, Theodor Fontane, Brandenburg, Germany; 3 Department of Surgery, Kingston Health Sciences Center, Queen’s University, Kingston, ON Canada; 4 Hamad General Hospital, Hamad Medical Corporation, Doha, Qatar

**Keywords:** Hernia, Learning curve, Shouldice repair, Shouldice hospital

## Abstract

**Purpose:**

The aim of the study was to evaluate operative time and postoperative complications of 4 post-training specialized surgeons.

**Methods:**

This was a pilot retrospective chart review to determine the learning curve of a Shouldice primary inguinal hernia repair (Shouldice Repair) of 4 post-training specialized surgeons, at the Shouldice Hospital. The first 300 Shouldice Repairs (early learning block) were compared to their 900-1,000 repairs as the primary operating surgeon (late learning block). Data was collected from the hospital’s database. The learning curve was examined using cumulative sum analysis (CUSUM).

**Results:**

During the early learning block cases, the surgeons had a mean operating time of 59.2 ± 11.2 min. The late learning block cases had significantly reduced operative time (53.4 ± 10.5 min, *p* = 0.001). According to the CUSUM analysis all four surgeons had a plateau after 78 to 88 operations in terms of operative time. A nonsignificant reduction in the rate of reported recurrences (*n* = 16 vs. *n* = 0) and surgical site occurrences (haematoma, seroma, infection; *n* = 27 vs. *n* = 2) was found between the early and late learning block cases.

**Conclusion:**

The operating time plateaued after 78–88 Shouldice Repairs for the 4 surgeons trained and working at the Shouldice Hospital. A nonsignificant trend towards fewer complications were noted among late learning block cases.

**Supplementary Information:**

The online version contains supplementary material available at 10.1007/s10029-024-03252-0.

## Introduction

The Shouldice primary inguinal hernia repair is a well-known and documented surgical repair [1, 2], and considered to be the gold standard non-mesh hernia repair [3]. The results in the literature for a Shouldice Repair vary greatly [2, 4] and may be due to a lack of accuracy when performing the repair [4, 5] and/or due to a lack of surgical volume and experience [6, 7]. An additional contributing factor could be that the Shouldice Repair is not principally taught to surgical trainees and therefore the learning curve is not well known outside of the Shouldice Hospital.

When learning a new skill or technique, a surgeon’s performance and development can be assessed when looking at individual and group learning curves Learning curves can be evaluated by determining the minimum number of procedures it takes trainees to reach similar outcomes as highly experienced surgeons [8]. Two publications on the learning curve for the Lichtenstein (4 surgeons, 400 cases) [8] and the transabdominal preperitoneal (TAPP) (unknown surgeons, 362 cases) hernia repairs have used operating times as the main outcome measure of a learning curve [9].

The learning curve for the Shouldice Repair at the Shouldice Hospital has not been formally examined. Therefore, the purpose of this study was to explore the learning curve of a Shouldice Repair done at the Shouldice Hospital.

## Methods

### Ethics

This study was approved by the Lakeridge Health Research Ethics Board.

### Study design

This was a retrospective chart review to collect information on the learning curve of a Shouldice primary inguinal hernia repair (referred to as Shouldice Repair in this study), at the Shouldice Hospital.

The learning curve was analyzed by using operating time which has also been done for Lichtenstein and similar research in TAPP repairs [8, 9, 10].

### Study objectives

1. To compare operative time between a surgeons first 300 Shouldice primary inguinal hernia repairs and their 900-1000th.
2. To examine if the operative time reaches a plateau during the early or late learning block.
3. To review learning curve and postoperative complications.

The parameter of 300 cases (considered the early learning block) was used as a parameter because the 2018 HerniaSurge guidelines state, “*Although no studies exist on a comparison of the learning curves of the different non-mesh techniques*,* the HerniaSurge group agrees that the Shouldice technique is not easy to learn. In The Shouldice Hospital*,* surgeons are only considered qualified after 300 cases!”* [3]. The late learning block, 900-1,000th Shouldice Repair for primary inguinal hernia, was chosen based on a previous publication from the Shouldice Hospital, “*These individuals were deemed as experts and invited to participate if they had each performed at least 1000 repairs*.” [2].

Postoperative complications included recurrences, hematomas, seromas and wound infections. This information was found during chart review, through follow-up notes in the patient’s chart. As there was no systematic follow-up, we did not measure time lapse to complications. KEY WORDS: Hernia, Learning curve, Shouldice repair, Shouldice hospital 

### Study population

The study population consisted of four surgeons who worked at the hospital in 2023, were hired within the last 10 years, and performed ≥ 1,000 primary inguinal hernia repairs. The operations where the surgeon was not the primary operating surgeon, i.e. the surgeon was labelled as the assistant or records showed the operation as done by both the primary and assistant surgeons (in training or training another) were excluded.

The patient data included a population of male and female patients accepted for surgery at the Shouldice Hospital, who had a Shouldice Repair. Patients were excluded if they underwent a mesh repair, repair of a scrotal hernia, non-Shouldice or modified Shouldice tissue repair, a hernia that was not an inguinal type, recurrent groin hernias, repairs with bowel resection, and/or and surgery was deemed an emergency.

### Definition of post-training specialized surgeons at the Shouldice Hospital

All surgeons undergo a training period, these cases were not included in the study. During this training period the surgeons had highly experienced senior surgeons acting as the assistant or teacher. Surgeons are finished training upon the chief surgeon’s approval. After this approval the newly independent surgeon operates without surgical assistance from senior surgeons. Once a surgeon obtains this approval they are considered as an independent post-training specialized surgeons.

### Data collection

Data was collected from the hospital’s database, records, as well as from a manual review of operative notes and charts. No follow up took place, postoperative outcomes were collected through recheck notes in the patient charts. Data was collected as close as possible to gather the approximate first consecutive 300 cases (early learning block operations) and 900-1000th cases (late learning block operations) where the surgeon was the primary operating surgeon. Hospital management was contacted for employment records, which were used to gather information on the surgeons training period, when surgeons are in “training” (assistant surgeons) versus operating independently. Additionally, a short questionnaire was emailed to the surgeons to gather data on previous surgical experience. The surgeons were asked about their experience prior to starting at the Shouldice Hospital (how many years of surgical experience they had, roughly how many inguinal hernia operations completed, and how many Shouldice Repairs had been done). Data was also collected on the number of operations each surgeon had done prior to their first operation as the primary operating surgeon (Shouldice Experience on primary inguinal hernias).

### Data analysis

Data analysis included descriptive statistics for frequency/percentage of the population as well as mean and standard deviations. The patient characteristics were computed for each trainee and compared univariably. Additional analysis included a multivariate analysis to determine if hernia size is a risk factor for longer operating time, as well as analysis to determine homogenous populations (comparing BMI, age, and gender). Sample size was not calculated as this was a pilot project.

Based on the initial 300 surgeries, the individual learning rates of each surgeon were determined. Descriptions of differences between individual learning curves were provided. In order to obtain a reliable estimate, an overall learning curve (pooled estimate) was determined, based on the geometric average of individual performance. The learning curve was examined using cumulative sum analysis (CUSUM). CUSUM analysis identifies differences between the raw data from each individual case and the mean value of the group. These deviations are then accumulated sequentially, allowing for the detection of trends, shifts, and turning points (known as trend transitions). Therefore, the curves move upward for longer operating times and increasing cases of postoperative complications. A plateau in the learning curve occurs when the CUSUM values level off or show minimal change.

Due to the nature of observational data, there is a possibility of an uneven distribution of patients with high complication risks among consecutive series or between surgeons. This can lead to bias, resulting in overestimated operative time and complication rates unrelated to the surgeon’s level of surgical experience during periods when high-risk patients are densely distributed. To address this possible bias, a risk-adjusted cumulative sum analysis (RA-CUSUM) was performed. Therefore, predictive factors for surgical duration and occurrence of postoperative complication were identified through a multivariate analysis. Linear regression models were employed for operating times and logistic regression models for complication occurrences. For multivariate regression modelling adjustment, the following variables were incorporated: age, BMI, sex, ASA score, hernia size, hernia location, number of comorbidities (diabetes, dyslipidemia, hypertension, coronary heart disease, deep vein thrombosis, stroke, transient ischemic attach, myocardial infarction, perivascular disease), and smoking. The multivariable models were employed to estimate the impact of the variables on the outcome. Accordingly, for each case with its respective value combinations, an expected operating time and a probability of the occurrence of a complication were calculated (expected value) using the multivariable regression models. The observed operating time or presence of a complication (coded as 1 for yes and 0 for no) was divided by the expected value and ratios above 1 indicate inferior performance while values below 1 indicate superior performance. Further details of the RA-CUSUM procedure, along with the mathematical details, are described elsewhere [10]. In principle, the CUSUM and RA-CUSUM line rises for longer operating times and more complications but falls for shorter operating times and fewer complications. High and low peaks within the RA-CUSUM curve are utilized to identify and differentiate possible learning phases. The shape of the learning curve in combination with the location of turning points may give hints for an optimal mentorship period. Data analysis was performed with R 4.2.3 (R Foundation, Vienna, Austria). Significance threshold was set to 0.05 for all analyses.

## Results

Four surgeons met the inclusion criteria and data was collected and analyzed.

Surgeon A had 14 years of surgical experience, performed 100 hernia repairs (Shouldice Repairs: *n* = 5) before being employed at the Shouldice Hospital. Surgeon A got assistance in 66 surgeries from a senior surgeon at Shouldice Hospital before getting chiefs approval. At that point Surgeon A was considered an independent post-training specialized surgeon.

Surgeon B had no surgical experience. Surgeon B got assistance in 874 surgeries from a senior surgeon at Shouldice Hospital before getting chiefs approval. At that point Surgeon B was considered an independent post-training specialized surgeon.

Surgeon C had 25 years of surgical experience, performed 300 hernia repairs (Shouldice Repairs: *n* = 20–30) before being employed at the Shouldice Hospital. Surgeon C got assistance in 72 surgeries from a senior surgeon at Shouldice Hospital before getting chiefs approval. At that point Surgeon C was considered an independent post-training specialized surgeon.

Surgeon D had 33 years of surgical experience, did not perform any hernia surgeries prior to his employment. Surgeon D got assistance in 188 surgeries from a senior surgeon at Shouldice Hospital before getting chiefs approval. At that point Surgeon D was considered an independent post-training specialized surgeon.

### Univariate analysis on baseline and perioperative characteristics

**Fig. 1:**
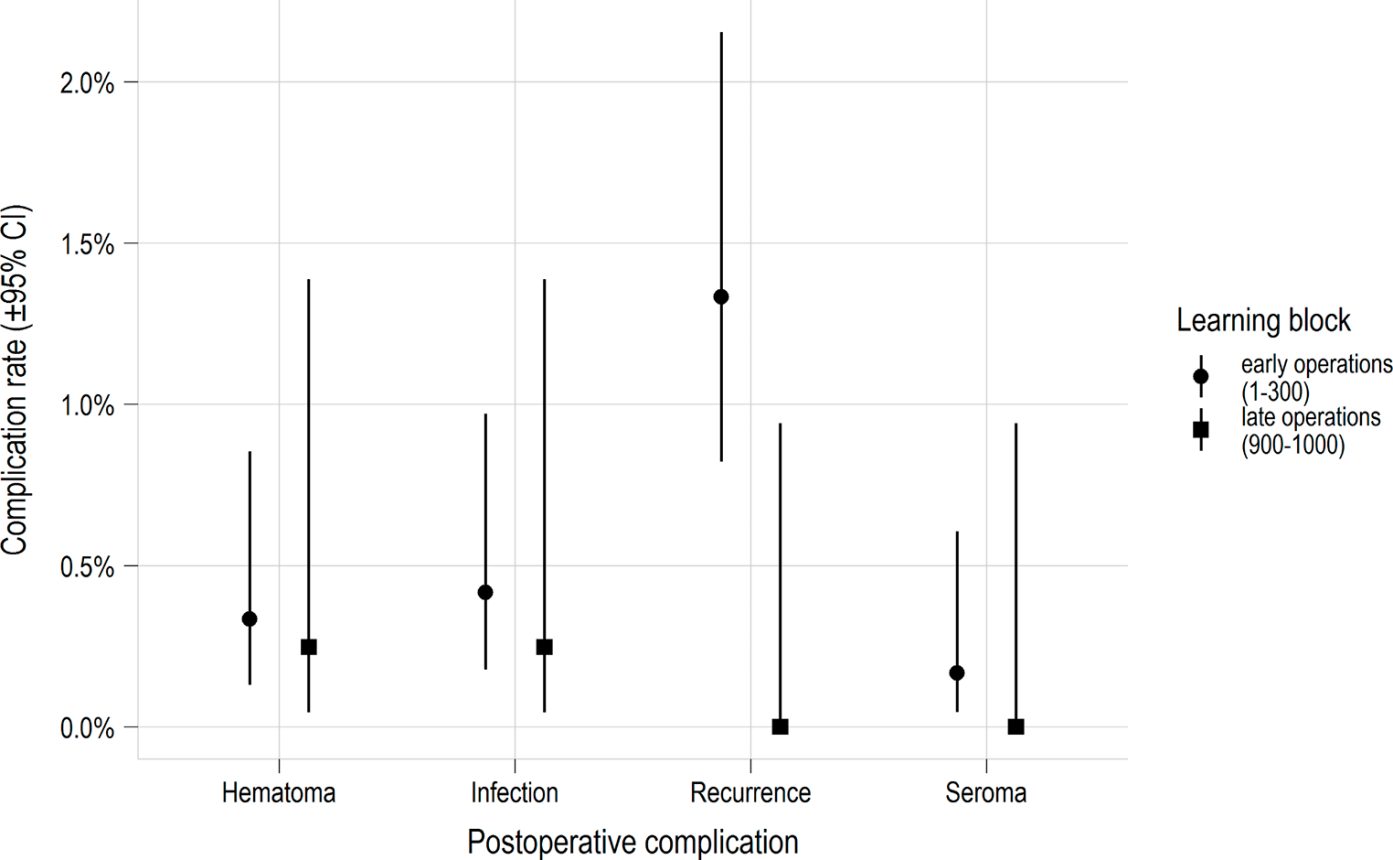
Postoperative complications comparing the early (0-300) and late (900–1000) learning blocks. A nonsignificant trend towards fewer complications were noted among late learning block cases (*n* = 27 vs. *n* = 2)

The descriptive results comparing patient, operation, and outcomes by surgeon are presented in the supplemental material (Table S1). The results comparing the characteristics collected from the early vs. late learning block cases are presented in Table 1. The complication rates, stratified by each learning block, are depicted in Fig. 1.

**Table 1:** Patient demographics, operation characteristics, and postoperative outcomes by early and late cases

	Early cases *N* = 1,200	Late cases *N* = 404	*p*-value
Age (years)	57.6 ± 14.7[16–91]	58.9 ± 14.1[18–88]	0.2
Body Mass Index	24.8 ± 2.4[16.5–33.4]	25.0 ± 2.2[18.3–30.4]	0.4
Gender			0.6
Male	1146 (95.5)	389 (96.3)	
Female	54 (4.5)	15 (3.7)	
Smoker	17(1.4)	5(1.2)	> 0.9
ASA			< 0.001
1	412(34.7)	90(27.1)	
2	568(47.8)	142(42.8)	
3	207(17.4)	100(30.1)	
4	1(0.1)	0(0)	
Number of Common Comorbidities*			0.2
0	795(66.4)	201(60.4)	
1	248(20.7)	72(21.6)	
2	107(8.9)	41(12.3)	
3	42(3.5)	16(4.8)	
4	6(0.5)	3(0.9)	
Hernia Side			0.3
Right	697(58.1)	222(55.0)	
Left	503(41.9)	182(45.0)	
Hernia Size			0.9
Small	244(20.4)	78(19.3)	
Medium	644(53.7)	223(55.2)	
Large	311(25.9)	103(25.5)	
Inguinal Hernia Type			0.5
Indirect	681(56.8)	224(55.4)	
Direct	379(31.6)	124(30.7)	
Both	140(11.7)	56(13.9)	
Associated Hernias**			0.3
Interstitial	13(1.1)	6(1.5)	
Spigelian	2(0.17)	0(0.0)	
Femoral	1(0.08)	1(0.25)	
Length of Operation	59.2 ± 11.2[24–133]	53.4 ± 10.5[27–107]	< 0.001
Postoperative Complications			
Recurrence#	16	0	0.070
Infection***	5	1	> 0.9
Hematoma	4	1	> 0.9
Seroma	2	0	> 0.9

Notes Data is presented as mean ± standard deviation [range] or incidence (percent, %). There was missing data on ASA for 84 patients, comorbidities for 73 patients, and 1 hernia size was unknown. *The common comorbidities included diabetes, dyslipidemia, hypertension, coronary heart disease, deep vein thrombosis, stroke, transient ischemic attack, myocardial infarction, perivascular disease, stents, angina, angioplasty, atrial fibrillation, Coronary artery bypass grafting, ischemic heart disease, neutropenia, chronic lymphocytic leukemia, obstructive sleep apnea, cardiomyopathy (hyper), arteriosclerosis. ** Interstitial hernias were either repaired with sutures or reduced, spigelian hernia was repaired with a tissue repair, and the femoral hernias by complete/total groin repair. *** No SSI grading took place but there were no reports of deep organ involvement (GradeIII)

### Descriptive analysis on learning curve

The average operative length for Surgeon A was 63.6 (12.4) minutes for cases 1-100, 59.4 (9.5) minutes for cases 101–200, 58.4 (9.5) minutes for the cases 201–300, and 54.7 (12.0) minutes for cases 900-1,000. The average length of operation for Surgeon B was 66.5 (14.1) minutes for cases 1-100, 60.2 (10) minutes for cases 101–200, 60.7 (10) minutes for cases 201–300, and 58 (8) minutes for cases 900-1,000. The average length of operation for Surgeon C was 61.9 (11.3) minutes for cases 1-100, 57.7 (8.8) minutes for cases 101–200, 55.5 (9.5) minutes for cases 201–300, and 47.1 (7.5) minutes for cases 900-1,000. The average length of operation for Surgeon D was 57.6. (8.6) minutes for cases 1-100, 54.9 (12.5) minutes for cases 101–200, 54.1 (10.7) minutes for cases 201–300, and 54.0 (10.8) minutes for cases 900–1000 (Figure S1-S2). On average, it took 1014 days (2.8 years, 2.5–3.4 years) to achieve 1000 Shouldice repairs for primary inguinal hernias.

### CUSUM analysis on operating time

The slope of the CUSUM curve is positive from cases 1–86, reaching a plateau between cases 87–165, and negative from case 166 onwards. These sections of the CUSUM graph correspond to the learning, competency, and mastery phases. All four surgeons reached a plateau in operating time after 78 to 88 operations.

### RA-CUSUM analysis on operating time

**Fig. 2:**
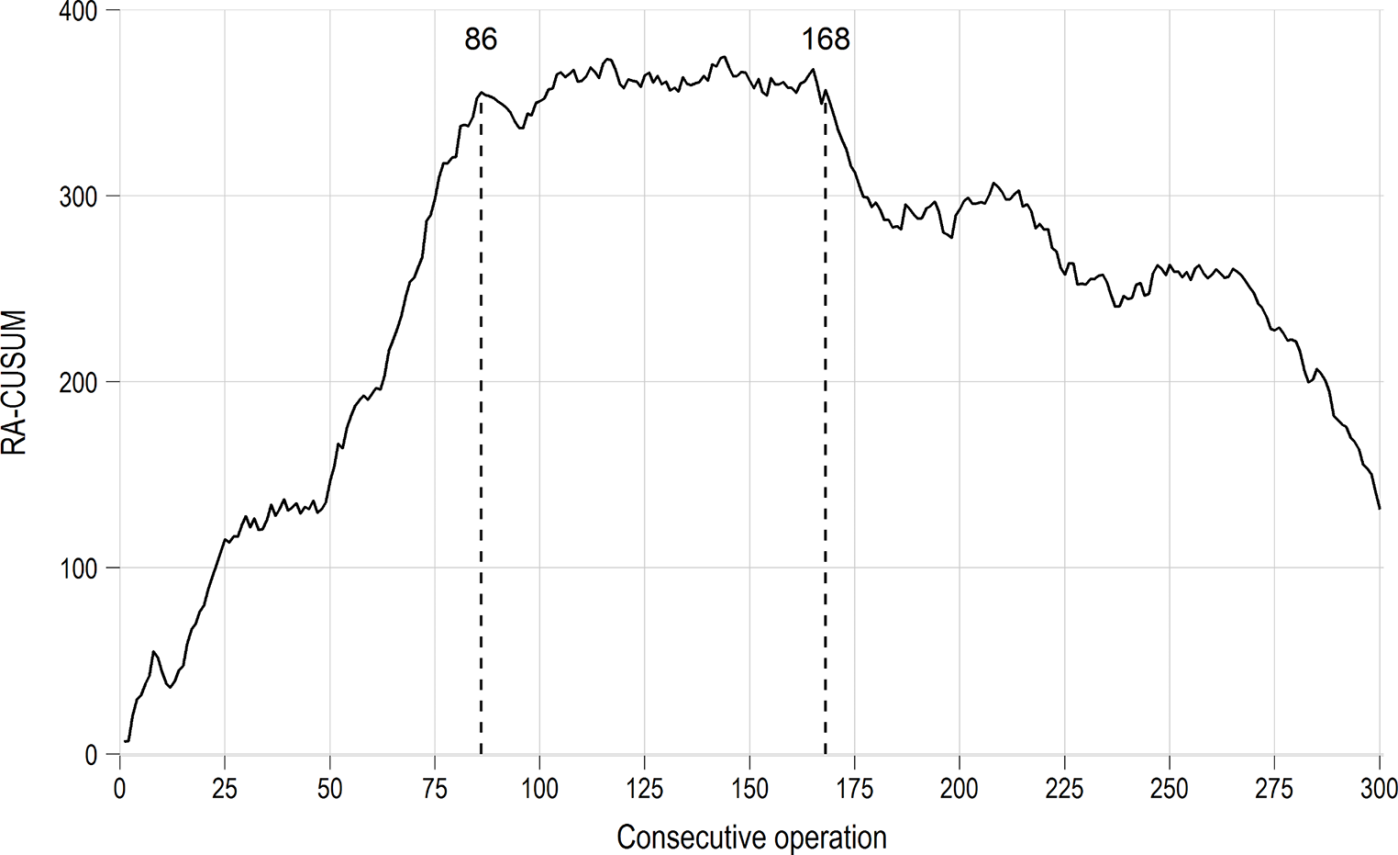
RA-CUSUM analysis of operating time. CUSUM analysis identifies differences between the raw data from each individual case and the mean value of the group. These deviations are then accumulated sequentially, allowing for the detection of trends, shifts, and turning points (known as trend transitions). The consecutive series of cases 1 to 300 is plotted on the x-axis from left to right. The y-axis depicts the difference between the cumulative expected operating time and the cumulative actual observed operating time (minutes). To explain further: compared to 25 cases with an average operation time, the first 25 took around 100 min longer in total. A multivariable regression model for operating time was performed to calculate the expected operating time, and includes sex, age, smoking, BMI, ASA, hernia location and size, number of comorbidities and suture material. The curves move upward for longer operating times and downward for shorter operating times. The slope of the RA-CUSUM curve is positive from cases 1–86, then reaches a plateau between cases 87–168, and is negative from case 168 onwards

The univariable effects of the covariates on operating time, as well as the results of the multivariable regression model are summarized in supplementary Table S2. Factors that substantially and significantly increased operating time in multivariable analysis were repair of femoral hernia (tissue repair) (+ 21.0 min, *p* = 0.003), large hernia size (+ 8.3 min, *p* < 0.001), male sex (+ 7.9 min, *p* < 0.001), and use of wire as suture material (+ 5.2 min, *p* < 0.001). Based on the regression model, the pooled risk-adjusted CUSUM curve was calculated (Fig. 2).

The learning phase thresholds are almost the same, as obtained in the adjusted CUSUM curve. The individual learning curves of each surgeon are depicted in supplementary Figure S1-3.

### Postoperative complications

The 27 postoperative complications (Recurrence: *n* = 16, Infection: *n* = 5, Hematoma: *n* = 4, Seroma: *n* = 2) were almost equally distributed among the first 300 operations. The logistic regression analysis identified only smoking as a significant patient risk factor (Odds ratio = 14.9, *p* < 0.001, Table S3).

## Discussion

The updated international HerniaSurge guidelines had stated that the Shouldice Repair is the best non-mesh recommended technique for primary inguinal hernias, however, the learning curve has not yet been reported [11]. According to the present findings, after 78 to 88 Shouldice Repairs by a fully trained surgeon, who has been trained by an experienced senior surgeon at the Shouldice Hospital, operative time plateaus.

The operating time and the learning curve are important when it comes to the introduction of a surgical approach. The open anterior mesh repair (mainly Lichtenstein repair) is considered the optimal open mesh-based repair approach for treating inguinal hernias, and the learning curve has been described as short [3]. To that, Bladin et al. (2022) published learning curve data from the Swedish Hernia Register (*n* = 38,845 repairs). Among the 663 surgeons the operation time decreased with increasing number of performed procedures. The average duration of surgery was 79 (± 26) minutes for the first 15 procedures and 60 (± 23) minutes after 241 procedures [12]. Merola et al. (2019) reported, that from the beginning of surgical residency 37–42 Lichtenstein repairs are needed to reach the learning curve. The authors reviewed data from 700 patients and compared 20 cases of a trainee to an experienced surgeon. The mean operating time ranged from 39.95 ± 8.9 min to 48.30 ± 7.89 min [8]. In the present study during the early learning block cases, the surgeons had a mean operating time of 59.2 (± 11.2) minutes. After approximately 78 to 88 cases the operative time reached a plateau (Figs. 1, 2 and S3). In comparison, the learning curve is flatter than the published curves of Bladin et al. (2022) and Merola et al. (2019) [8, 12]. These results are consistent with the general assumption in the hernia community that the Shouldice Repair is more demanding and takes longer to learn. On the other hand, the supervised learning phase was not included in our study. Hence, the comparison of our data with those mentioned above are only possible to a certain extent.

As these factors are also of economic interest, the operating time of the Shouldice Repair may compete with those of the open anterior mesh repair (mainly Lichtenstein repair) and the TAPP repair. Surgeon B did not have prior surgical experience and the average operating time for their first 10 cases was on average below 65 min. With respect to the pooled data, the operation time of the late learning block cases in this study (900-1,000) were only 2 min longer (53.4 ± 10.5 min) when compared to performing an open anterior mesh repair by an expert (about 52 min) [8]. On the other hand, it could be argued that the operative time of an open anterior mesh repair (mainly Lichtenstein repair) will be even lower after 900 cases as in our study. Bladin et al. (2022) documented the learning curve for only about 240 procedures from the Swedish Hernia Registry [12]. A meaningful comparison would require data from a Lichtenstein Clinic, then numbers could be comparable to those of the Shouldice Hospital. In addition, the operative time was not worse for an open hernia repair compared to TAPP repair. Brucci et al. (2023) published a median operative time of 55 min, while the median operative time for surgeons in training was 93 min. The authors analysed 487 hernia repairs, with 319 procedures performed by experienced surgeons and 168 procedures performed by junior surgeons [9]. Other authors came to similar conclusions [13].

In general, a decreasing operative time is only, to a certain extent, correlated with a decreasing rate of surgical site occurrences and recurrences. From our perspective, a surgeon is considered an expert when they have low surgical site occurrences and recurrences. We can assume that some specialized and experienced surgeons will always have longer operating times due to personal style, but have a very low complication rate. There was a trend towards a reduction in reported recurrences between the early and late learning block cases, however this was not significant (1.3–0%, *p* = 0.07). Without significance the rate of surgical site occurrences was also lower (Table 1; Fig. 1). In addition, the CUSUM-Analysis on postoperative complications showed that the rate of postoperative complications (including recurrences) decreased in accordance with the decrease of the operating time (Figs. 1 and S3). Based on these findings it can be assumed that up to 1,000 Shouldice Repairs are needed, with previous specialized training, to be considered an expert. This is in accordance with statements from the guidelines [3] and previous publications [2]. However, more research is needed to evaluate this assumption.

The average operating time increases again after reaching the lowest point after 930 Shouldice Repairs. These results could reflect the fact that the complexity of the cases is increasing, more experienced surgeons are operating on more complex cases. In addition, the ASA score of patients in the late learning block cases was significantly higher as well as these individuals were older (Table 1). To our knowledge, similar results have not been published in the field of hernia surgery discussing increased operative times in a group of specialized surgeons. The most closely related publications by Bladin et al. (2022) and Merola et al. (2019) did not follow the learning curve of 1,000 cases in open anterior mesh repair. Bladin et al. (2022) observed approximately the first 250 cases [12] and Merola et al. (2019) identified the first 100 operations [8].

The frequency of the surgical site occurrence of (seroma, infection, and haematoma) and recurrences were determined by a review of the medical records. Patients are asked to report back any complications after surgery. When our research team conducted and published the research project entitled *Polypropylene vs. stainless-steel wire suture: short-term recurrence rate after Shouldice primary inguinal hernia repair*,* a non-inferior analysis among 1120 patients. A case–control study* in 2023, all patients were called by phone to have their complications and recurrences assessed [14] We found that 2/3 of our patients with a postoperative recurrence and SSOs reported it [14]. Although there was no systematic prospective follow-up, the recurrence and complication rates may be largely reliable. On the other hand, no adjustment was made for time elapsed since surgery, which could be a possible bias, as the earlier repairs have had a longer time to recur.

From our point of view, it should be considered that the learning curve can be influenced by the surgical assistants and scrub nurses. The Shouldice Hospital has a high case load, with 25 to 30 operations per day. A new surgeon has the chance to be trained through a series of Shouldice Repairs instead of having only one or two cases per week. For this reason, the results presented may not be reproducible elsewhere.

### Study limitations

For the pooled data, the performances of only 4 male surgeons with different levels of experience were used. A sufficient gender distribution, an equal level of experience, and a higher number of surgeons would have led to more clear results. On the other hand, the pattern of the learning curve is similar for all 4 surgeons. The results therefore appear to apply to both experienced and inexperienced surgeons.

As there was no systematic follow-up, surgical site infections and recurrences were only reported based on patients who reported complications. No exact time periods can be given. It can be assumed that some complications were treated in other hospitals without informing the Shouldice Hospital. For that reason, these variables were not used as a primary endpoint.

## Conclusion

The operating time plateaued after 78–88 Shouldice Repairs for the 4 surgeons trained and working at the Shouldice Hospital. These results may encourage colleagues to perform the procedure, as that the learning curve to reduce operating time can be performed in a reasonable time period. However, the learning curve to reduce postoperative complications remains unknown.

## Electronic supplementary material

Below is the link to the electronic supplementary material.

Supplementary Material 1

## Data Availability

Not applicable.
