# Supplementary material for: The learning curve for the Shouldice Repair: a pilot analysis of post-training specialized surgeons at the Shouldice Hospital
Source: Hernia. 2025 Jan 23;29(1):70. doi: 10.1007/s10029-024-03252-0 (PMC11757887; doi:10.1007/s10029-024-03252-0)
Supplement: Supplementary file 1 — Supplementary Material 1 [file 10029_2024_3252_MOESM1_ESM.docx]

**Supplemental Material**

**Table S1** Patient demographics, operation characteristics, and postoperative outcomes by surgeon

|  | **Surgeon A** | **Surgeon B** | **Surgeon C** | **Surgeon D** | **p-value** |
| --- | --- | --- | --- | --- | --- |
| Age (years) | 60.9 ± 14  [19–89] | 55.0 ± 15.6  [16–86] | 57.8 ± 13.9  [16–91] | 58.1 ± 14.2  [17–88] | < 0.001 |
| Body Mass Index | 25.0 ± 2.4  [16.5–33.4] | 24.4 ± 2.2  [18.7–32.8] | 25.0 ± 2.5  [18.1–31.8] | 25.2 ± 2.2  [19.0-32.1] | < 0.001 |
| Gender |  |  |  |  | 0.5 |
| Male | 381(95) | 389(97) | 382(95.3) | 383(95.5) |  |
| Female | 20(5) | 12(3) | 19(4.7) | 18(4.5) |  |
| Smoker | 3(0.7) | 7(1.7) | 6(1.5) | 6(1.5) | 0.6 |
| ASA |  |  |  |  | < 0.001 |
| 1 | 72(18) | 167(52.2) | 134(33.7) | 129(32.2) |  |
| 2 | 225(56.1) | 128(40) | 182(45.7) | 175(43.6) |  |
| 3 | 104(25.9) | 25(7.8) | 81(20.4) | 97(24.2) |  |
| 4 | 0(0) | 0(0) | 1(0.3) | 0(0) |  |
| Number of Common Comorbidities* |  |  |  |  | 0.002 |
| 0 | 229(57.1) | 237(72.3) | 271(67.6) | 259(64.6) |  |
| 1 | 95(23.7) | 61(18.6) | 75(18.7) | 89(22.2) |  |
| 2 | 50(12.5) | 18(5.5) | 41(10.2) | 39(9.7) |  |
| 3 | 21(5.2) | 11(3.4) | 12(3) | 14(3.5) |  |
| 4 | 6(1.5) | 1(0.3) | 2(0.5) | 0(0) |  |
| Hernia Side |  |  |  |  | 0.2 |
| Right | 245(61.1) | 220(54.9) | 221(55.1) | 233(58.1) |  |
| Left | 156(38.9) | 181(45.1) | 180(44.9) | 168(41.9) |  |
| Hernia Size |  |  |  |  | < 0.001 |
| Small | 48(12) | 68(17) | 95(23.7) | 111(27.7) |  |
| Medium | 185(46.1) | 263(65.8) | 216(53.9) | 203(50.6) |  |
| Large | 168(41.9) | 69(17.3) | 90(22.4) | 87(21.7) |  |
| Inguinal Hernia Type |  |  |  |  | < 0.001 |
| Indirect | 249(62.1) | 203(50.6) | 236(58.9) | 217(54.1) |  |
| Direct | 124(30.9) | 130(32.4) | 130(32.4) | 119(29.7) |  |
| Both | 28(7) | 68(17) | 35(8.7) | 65(16.2) |  |
| Associated Hernias** |  |  |  |  | 0.008 |
| Interstitial | 2(0.5) | 9(2.2) | 1(0.2) | 7(1.7) |  |
| Spigelian | 0(0) | 0(0) | 1(0.2) | 1(0.2) |  |
| Femoral | 0(0) | 1(0.2) | 0(0) | 1(0.2) |  |
| Length of Operation | 59.0 ± 11.4  [32–107] | 61.3 ± 11.2  [34–133] | 55.5 ± 10.8  [27–105] | 55.1 ± 10.8  [24–105] | < 0.001 |
| Postoperative Complications |  |  |  |  |  |
| Recurrence | 4 | 0 | 3 | 9 | 0.024 |
| Infection | 2 | 3 | 1 | 0 | 0.2 |
| Hematoma | 1 | 1 | 0 | 3 | 0.3 |
| Seroma | 1 | 0 | 1 | 0 | 0.6 |

Notes: Data is presented as mean ± standard deviation [range] or incidence (percent, %). There was missing data on ASA for 84 patients, comorbidities for 73 patients, and 1 hernia size was unknown. *The common comorbidities included diabetes, dyslipidemia, hypertension, coronary heart disease, deep vein thrombosis, stroke, transient ischemic attack, myocardial infarction, perivascular disease, stents, angina, angioplasty, atrial fibrillation, coronary artery bypass grafting, ischemic heart disease, neutropenia, chronic lymphocytic leukemia, obstructive sleep apnea, cardiomyopathy(hyper), arteriosclerosis. ** Interstitial hernias were either repaired with sutures or reduced, spigelian hernia was repaired with a tissue repair, and the femoral hernias by complete/total groin repair.

**Table S2** Univariable and multivariable effects of the covariates on operating time

|  | **Unadjusted/Univariable Effects** | | |  |  | | **Adjusted/Multivariate Effects** | | |  |  | |
| --- | --- | --- | --- | --- | --- | --- | --- | --- | --- | --- | --- | --- |
|  | **Beta** | **95% CI^1^** | **p-value** | | | **Beta** | | **95% CI^1^** | **p-value** | | |  |
| Age (years) | -0.07 | -0.11,-0.03 | < 0.001 | | | -0.08 | | -0.13,-0.04 | < 0.001 | | |  |
| Gender |  |  |  | | |  | |  |  | | |  |
| Male |  |  |  | | |  | |  |  | | |  |
| Female | -8.74 | -11.37,-6.10 | < 0.001 | | | -6.77 | | -9.37,-4.17 | < 0.001 | | |  |
| Smoker |  |  |  | | |  | |  |  | | |  |
| No |  |  |  | | |  | |  |  | | |  |
| Yes | -0.57 | -5.23,4.09 | 0.8 | | | -1.76 | | -6.14,2.62 | 0.4 | | |  |
| Body Mass Index | 1.16 | 0.93,1.39 | < 0.001 | | | 0.91 | | 0.68,1.14 | < 0.001 | | |  |
| ASA |  |  |  | | |  | |  |  | | |  |
| 1 |  |  |  | | |  | |  |  | | |  |
| 2 | -0.63 | -1.91,0.67 | 0.3 | | | -0.33 | | -1.64,0.97 | 0.6 | | |  |
| 3 | -0.93 | -2.57,0.70 | 0.3 | | | -0.22 | | -2.09,1.66 | 0.8 | | |  |
| 4 | 5.94 | -15.99,27.88 | 0.6 | | | 7.28 | | -12.67,27.23 | 0.5 | | |  |
| Number of Common Comorbidities |  |  |  | | |  | |  |  | | |  |
| 0 |  |  |  | | |  | |  |  | | |  |
| 1 | 0.34 | -1.07,1.75 | 0.6 | | | 1.11 | | -0.35,2.57 | 0.13 | | |  |
| 2 | 0.41 | -1.52,2.35 | 0.7 | | | 1.15 | | -0.84,3.14 | 0.3 | | |  |
| 3 | -1.36 | -4.31,1.60 | 0.4 | | | -0.89 | | -3.88,2.10 | 0.6 | | |  |
| 4 | -2.29 | -9.64,5.06 | 0.5 | | | 0.13 | | -6.65,6.92 | > 0.9 | | |  |
| Hernia Side |  |  |  | | |  | |  |  | | |  |
| Right |  |  |  | | |  | |  |  | | |  |
| Left | 0.37 | -0.73,1.46 | 0.5 | | | 0.4 | | -0.64,1.43 | 0.5 | | |  |
| Hernia Size |  |  |  | | |  | |  |  | | |  |
| Small |  |  |  | | |  | |  |  | | |  |
| Medium | 2.20 | 0.83,3.56 | 0.002 | | | 1.80 | | 0.43,3.16 | 0.010 | | |  |
| Large | 8.59 | 7.01,10.17 | < 0.001 | | | 7.49 | | 5.89,9.09 | < 0.001 | | |  |
| Hernia Type |  |  |  | | |  | |  |  | | |  |
| Indirect |  |  |  | | |  | |  |  | | |  |
| Direct | -3.74 | -4.93,-2.54 | < 0.001 | | | -4.33 | | -5.48,-3.18 | < 0.001 | | |  |
| Both | -0.49 | -2.20,1.21 | 0.6 | | | -0.31 | | -1.96,1.34 | 0.7 | | |  |
| Associated Hernias/Repair |  |  |  | | |  | |  |  | | |  |
| None |  |  |  | | |  | |  |  | | |  |
| Interstitial/Suture | 3.55 | -2.73,9.84 | 0.3 | | | 5.99 | | -0.04,12.02 | 0.051 | | |  |
| Interstitial/Unknown | 5.35 | -9.99,20.69 | 0.5 | | | 6.09 | | -7.99,20.17 | 0.4 | | |  |
| Interstitial/Reduced | -1.75 | -11.49,7.99 | 0.7 | | | 1.19 | | -7.75,10.12 | 0.8 | | |  |
| Femoral/Total Groin Repair | 19.38 | 4.06,34.70 | 0.013 | | | 20.99 | | 6.95,35.03 | 0.003 | | |  |
| Spigelian/Tissue | 11.17 | -4.15,26.49 | 0.2 | | | 11.07 | | -3.10,25.24 | 0.13 | | |  |
| Unknown/Stitches between transversalis fascia and coopers | -5.57 | -27.24,16.09 | 0.6 | | | -4.89 | | -24.72,14.95 | 0.6 | | |  |
| Suture Material |  |  |  | | |  | |  |  | | |  |
| Wire |  |  |  | | |  | |  |  | | |  |
| Prolene | -4.27 | -6.71,-1.83 | < 0.001 | | | -5.76 | | -8.03,-3.49 | < 0.001 | | |  |

^1^ CI = Confidence Interval

**Table S3** Univariable and multivariable effects (logistic regression) of the covariates on post-operative complication

|  | **Unadjusted/Univariable Effects** | | |  |  | | **Adjusted/Multivariate Effects** | | |  |  | |
| --- | --- | --- | --- | --- | --- | --- | --- | --- | --- | --- | --- | --- |
|  | **OR** | **95% CI** | **p-value** | | | **OR** | | **95% CI** | **p-value** | | |  |
| Age (years) | 1.00 | 0.98,1.03 | 0.7 | | | 0.99 | | 0.9*6,1.02 | 0.6 | | |  |
| Gender |  |  |  | | |  | |  |  | | |  |
| Male |  |  |  | | |  | |  |  | | |  |
| Female | 1.73 | 0.40,7.44 | 0.5 | | | 2.64 | | 0.56,12.52 | 0.2 | | |  |
| Smoker |  |  |  | | |  | |  |  | | |  |
| No |  |  |  | | |  | |  |  | | |  |
| Yes | 14.62 | 4.57,46.75 | < 0.001 | | | 15.12 | | 4.23,54.00 | < 0.001 | | |  |
| Body Mass Index | 1.03 | 0.88,1.22 | 0.7 | | | 0.99 | | 0.84,1.16 | 0.9 | | |  |
| ASA |  |  |  | | |  | |  |  | | |  |
| 1 |  |  |  | | |  | |  |  | | |  |
| 2 | 0.95 | 0.38,2.33 | > 0.9 | | | 0.75 | | 0.26,2.15 | 0.6 | | |  |
| 3 | 1.65 | 0.61,4.45 | 0.3 | | | 1.15 | | 0.30,4.39 | 0.8 | | |  |
| Number of Common Comorbidities |  |  |  | | |  | |  |  | | |  |
| 0 |  |  |  | | |  | |  |  | | |  |
| 1 | 1.10 | 0.14,8.45 | > 0.9 | | | 0.82 | | 0.10,7.02 | 0.9 | | |  |
| 2 | 0.91 | 0.10,8.28 | > 0.9 | | | 0.86 | | 0.09,8.35 | 0.9 | | |  |
| 3 | 3.04 | 0.36,25.75 | 0.3 | | | 2.19 | | 0.22,21.75 | 0.5 | | |  |
| 4 | 2.57 | 0.23,29.08 | 0.4 | | | 1.97 | | 0.14,26.93 | 0.6 | | |  |
| Hernia Side |  |  |  | | |  | |  |  | | |  |
| Right |  |  |  | | |  | |  |  | | |  |
| Left | 1.17 | 0.55,2.48 | 0.7 | | | 1.21 | | 0.56,2.61 | 0.6 | | |  |
| Hernia Size |  |  |  | | |  | |  |  | | |  |
| Small |  |  |  | | |  | |  |  | | |  |
| Medium | 1.12 | 0.35,3.52 | 0.9 | | | 1.44 | | 0.43,4.81 | 0.6 | | |  |
| Large | 2.38 | 0.76,7.46 | 0.14 | | | 2.93 | | 0.84,10.20 | 0.092 | | |  |
| Hernia Type |  |  |  | | |  | |  |  | | |  |
| Indirect |  |  |  | | |  | |  |  | | |  |
| Direct | 1.39 | 0.61,3.21 | 0.4 | | | 1.49 | | 0.63,3.53 | 0.4 | | |  |
| Both | 1.80 | 0.63,5.11 | 0.3 | | | 1.57 | | 0.52,4.71 | 0.4 | | |  |
| Suture Material |  |  |  | | |  | |  |  | | |  |
| Wire |  |  |  | | |  | |  |  | | |  |
| Prolene | 0.54 | 0.07,4.15 | 0.6 | | | 0.47 | | 0.06,3.66 | 0.5 | | |  |

Notes: The factor “other hernias present and repaired” was not included as a predictor, as no complications occurred. OR = odds ratio. CI = confidence interval.


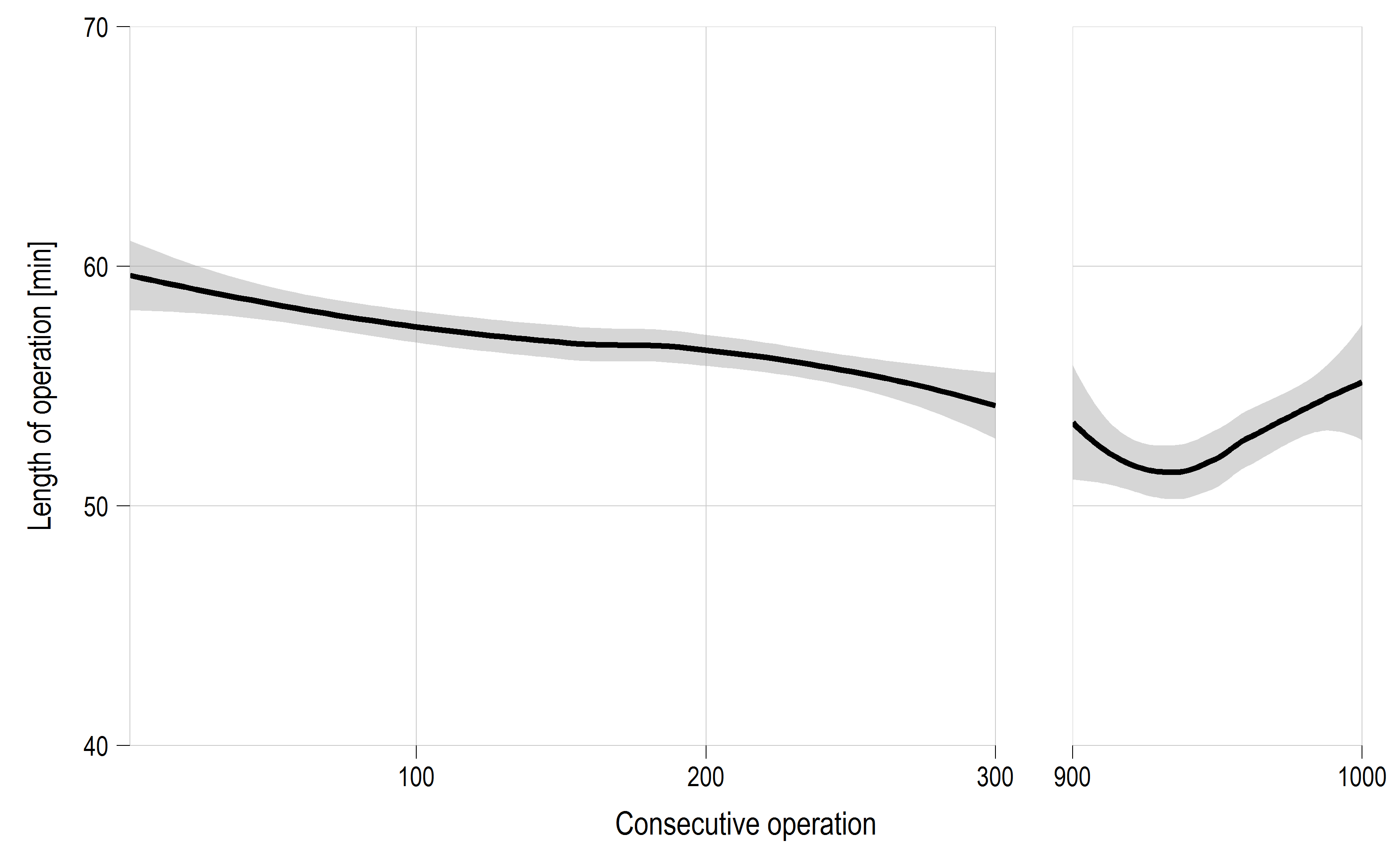


**Figure S1** Learning curve averaged across all four surgeons


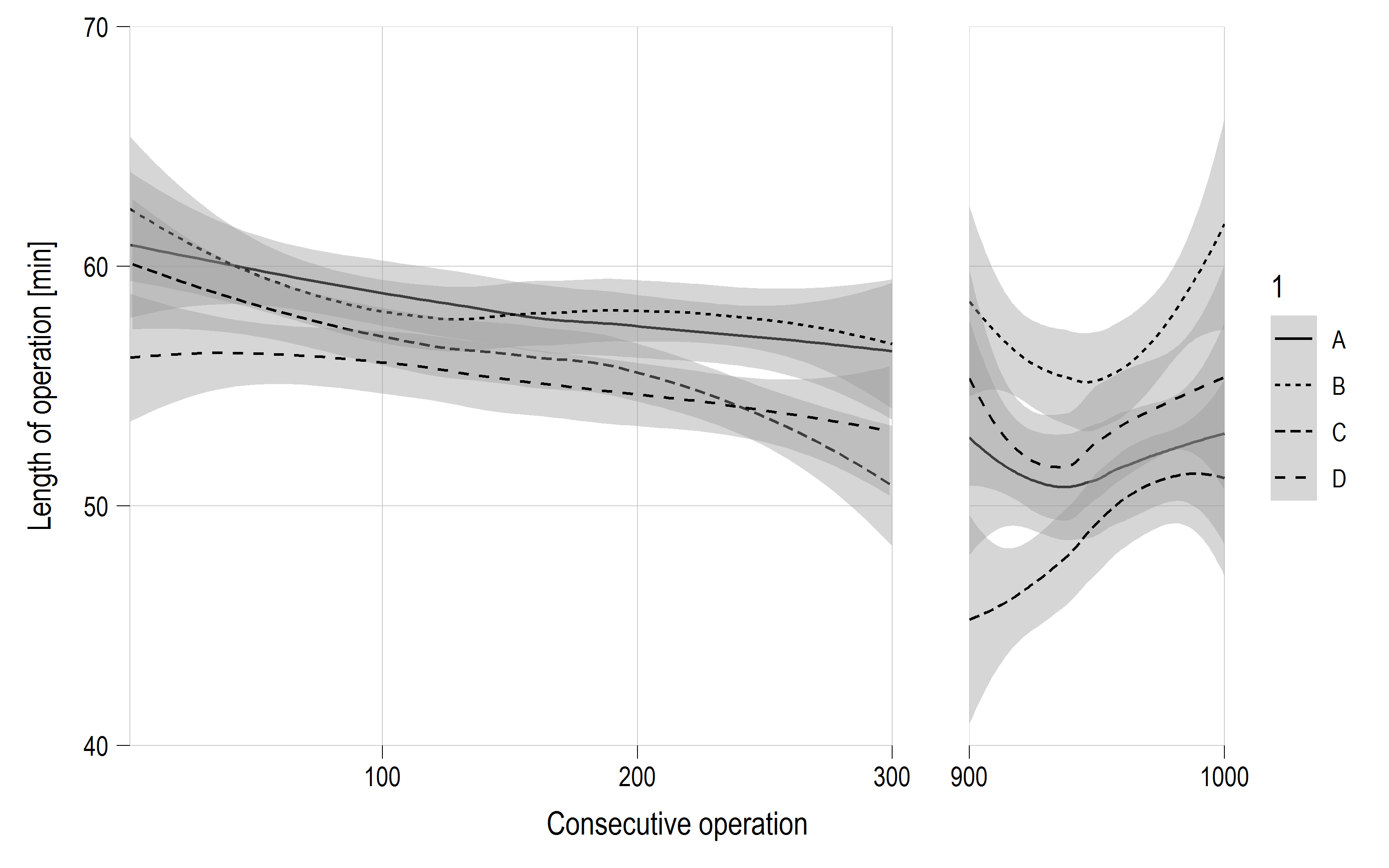


**Figure S2** CUSUM graph depicting learning phases for each surgeon


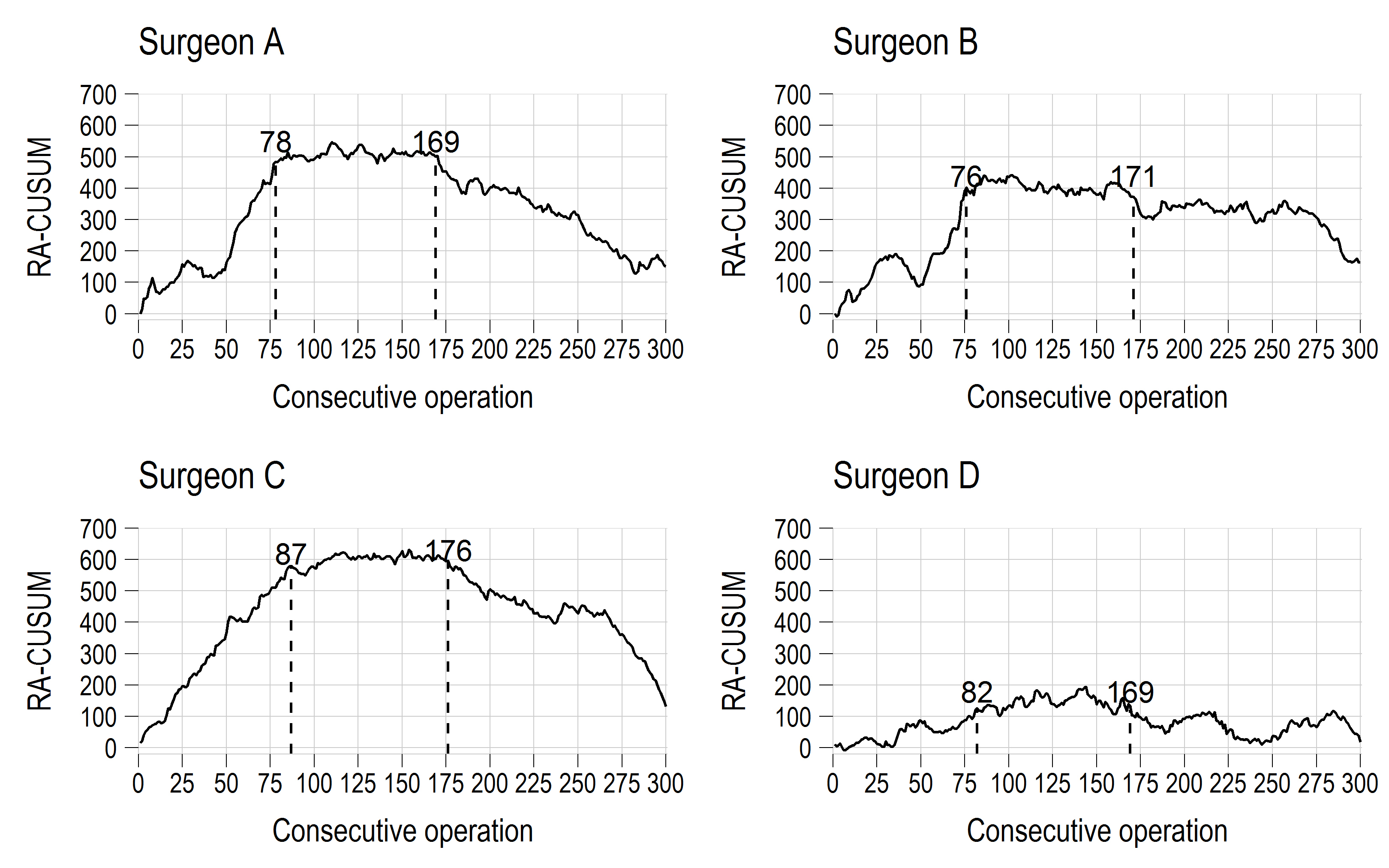


**Figure S3** RA-CUSUM graph depicting learning phases for each surgeon
